# *BRAF* Mutations in Patients with Non-Small Cell Lung Cancer: A Systematic Review and Meta-Analysis

**DOI:** 10.1371/journal.pone.0101354

**Published:** 2014-06-30

**Authors:** Dong Chen, Li-Qun Zhang, Jun-Fu Huang, Kai Liu, Zheng-Ran Chuai, Zhao Yang, Yun-Xia Wang, Da-Chuan Shi, Qian Liu, Qing Huang, Wei-Ling Fu

**Affiliations:** 1 Department of Laboratory Medicine, Southwest Hospital, Third Military Medical University, Chongqing, PR China; 2 Research Center for Nutrition and Food Safety, Institute of Military Preventive Medicine, Third Military Medical University, Chongqing, PR China; University General Hospital of Heraklion and Laboratory of Tumor Cell Biology, School of Medicine, University of Crete, Greece

## Abstract

**Background:**

*BRAF* mutations have been well described in non-small cell lung cancer (NSCLC) for several years, but the clinical features of patients harboring *BRAF* mutations are still not well described. We performed a meta-analysis to identify common clinical features in NSCLC patients carrying *BRAF* mutations.

**Methods:**

We identified clinical studies that examined the association between *BRAF* mutations and features of NSCLC within PubMed, Embase and ISI Science Citation Index database up to October 2013. The effect size of clinical features was estimated by odds ratios (ORs) with 95% confidence interval (CI) for each study, using a fixed-effects or random-effects model.

**Results:**

Ten studies with a total of 5599 NSCLC patients were included. There was a 3% (170/5599) *BRAF* mutation rate. *BRAF* mutations in NSCLC were significantly associated with adenocarcinomas (ADCs) (compared with non-ADCs, OR = 4.96, 95%CI = 2.29–10.75). There were no significant differences in gender, smoking and stage in patients with and without *BRAF* mutations. The *BRAF* ^V600E^ mutation was more frequent in women than non-*BRAF* ^V600E^ mutations (OR = 0.27, 95%CI = 0.12–0.59), and was closely related to never smokers (OR = 0.14, 95%CI = 0.05–0.42).

**Conclusions:**

These findings have important implications for the prediction of the NSCLC sub-types more accurately combined with other genetic changes.

## Introduction

Lung cancer is the leading cause of cancer death, annually resulting in more than one million deaths worldwide [1]. Approximately 85% of lung cancer patients have a histologic diagnosis of non-small cell lung cancer (NSCLC), and the overall 5-year survival rate is about 17% [2], [3]. Treatment decisions for patients with lung cancer have historically been based on tumour histology. One promising treatment strategy has focused on the further subdivision of NSCLC into clinically relevant molecular subsets. The classification schema was based on specific so-called driver mutations including activating mutations in the epidermal growth factor receptor (EGFR), *KRAS*, *BRAF*, *HER2*, *PIK3CA*, and others in frequencies exceeding 1% [4], [5]. Besides, Seidel et al. have successfully predicted each of the NSCLC sub-types by using a combination of immunohistochemistry and genomic markers [6].

*BRAF*, one of three members of the RAF kinase family: *A-RAF*, *BRAF*, *C-RAF*, belongs to the group of serine-threonine kinases and plays vital role in mitogen-activated protein kinase (MAPK) pathways [7], [8]. Mutations in *BRAF* has been found in different kinds of cancers, predominantly melanoma, metastatic colorectal cancer and papillary thyroid cancer. The frequency of mutation has been about 50%, 9%, and 45%, respectively [9]–[11]. *BRAF* mutations were found in 1–3% of NSCLCs [4].

The mutations in the above genes are closely related to specific demographic or clinicopathologic characteristics, including smoking habits, gender, clinical stage, and tumor histology [12], [13]. This information may be useful for the selection of patients for treatment with specific gene inhibitors. While *BRAF* mutations in NSCLC have been described for several years, the actual prevalence and clinical features of patients with NSCLC who harbor *BRAF* mutations are not well defined due to the relatively low number of patient cases investigated [14]–[16].

We performed a meta-analysis of a large number of lung tumors with *BRAF* mutations from published studies in order to quantitatively review the association between *BRAF* mutation and the demographic or clinicopathologic characteristics.

## Materials and Methods

### Publication search

We performed a systematic literature search in PubMed, EMBASE, and the Science Citation Index databases. The following search terms were used to identify relevant publications: “*BRAF*”, “*B-RAF*”, “non-small cell lung cancer”, “non-small cell lung carcinoma”, “non-small cell carcinoma”, “NSCLC”, “squamous-cell lung cancer”, “squamous-cell lung carcinoma”, “large-cell lung cancer”, “large-cell lung carcinoma”, “lung adenocarcinoma”. The literature search was limited to human studies. No limitations were placed on the language of publication or type of study. All eligible studies were retrieved, and their bibliographies were checked for other relevant publications. Review articles and bibliographies of other relevant studies identified were hand searched to find additional eligible studies.

### Inclusion criteria

Studies eligible for inclusion in this meta-analysis (1) were published as a full text in English. (2) the number of patients with *BRAF* mutations was more than 1; (3) the articles were involved with the association between *BRAF* and demographic or clinicopathologic features of NSCLC. When the same author or group reported results from the same patient population in more than one article, the most recent report or the most informative one was included.

### Data extraction

Information was carefully extracted from all eligible studies. The following data were collected from each study: first author’s name, year of publication, number of patients included, number of patients with *BRAF* mutations, number of patients with *BRAF* ^V600E^ mutations, screening methods, demographic and clinicopathologic characteristics of patients. Data extraction was done independently by two of the authors and discrepancies were resolved by consensus including a third author. All of the procedures conformed to the guidelines for the meta-analysis of observational studies in epidemiology [17].

### Statistical methods

We used RevMan (version 5) to calculate the summary odds ratios (ORs) with 95% confidence intervals (CIs), using a random or fixed effects model for all of the analyses. We assessed the heterogeneity of the studies using the chi-square test of heterogeneity and the I^2^ measure of inconsistency. Significant heterogeneity was defined as a chi-square test P value <0.10 or as an I^2^ measure >50% [18]. If ORs were found to have fine homogeneity (I^2^≤50%), a fixed effects model was used for secondary analysis. If not (I^2^>50%), a random-effects model was used. Sensitivity analysis was performed to examine the influence of each study on the pooled OR by serially omitting an individual study and pooling the remaining studies. Possible publication bias was evaluated by visual assessment of a funnel plot. Subgroup analyses were performed by ethnicity and number of *BRAF* mutations.

## Results

### Study Selection

**Figure 1:**
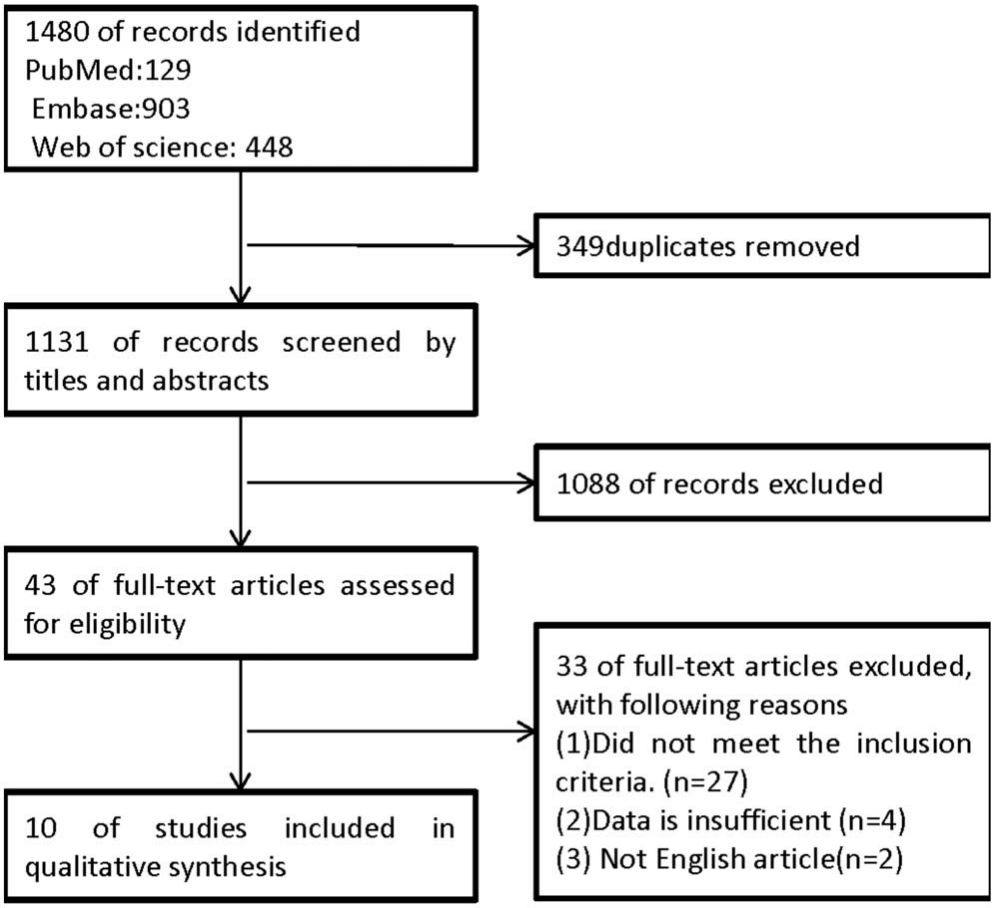
The flow chart for the selection of studies used in the meta-analysis.

A total of 1480 abstracts and titles were obtained through electronic searches. 349 records were excluded because of duplicates. The remaining 1131 records were screened by the titles and abstracts and 1088 studies were excluded. 43 full-text papers were deemed relevant and were examined in detail. 33 of these full-text articles were excluded (Figure 1). Ten studies met the inclusion criteria and were included in the meta-analysis.

### Study Characteristics

There were 5599 patients in the identified 10 studies [5], [14]–[16], [19]–[24]. Only 170 (3.0%) of these patients had *BRAF* mutations in the NSCLC tumors (Table 1). The earliest study was in November 2008 by Pratilas et al. [19], while the latest study was in August 2013 by Cardarella et al. [5]. The sample size ranged from 96 to 1046, with only one study over 1000 patients [16]. The incidence of *BRAF* mutations in individual studies ranged from 0.9% to 8.9%. Patients in four studies were Asian, five studies were Non-Asian, and one study consisted of a mixed population from 4 countries. Five studies screened for *BRAF* mutations using polymerase chain reaction (PCR) and direct sequencing. Three studies detected the *BRAF* mutations using the above methods plus matrix assisted laser desorption/ionization time of flight mass spectrometry (MALDI-TOF MS), single strand conformation polymorphism (SSCP) analysis, or high-resolution melting analysis (HRMA). The remaining two studies used the single methods MALDI-TOF MS or HRMA.

**Table 1:** Characteristics of the studies included in the meta-analysis.

First author	Year	Source of Pts	Methods	No. of Pts	MutBRAF (%)	Female (%)	Smokers (%)	ADC (%)	Stage III/IV (%)
Pratilas^18^	2008	4 countries	PCR+SEQ/MALDI-TOF MS	916	17(1.9)	577(63.0)	614(67.0)	623(68.0)	NA
Schmid^13^	2009	Austria	PCR+SEQ	96	2(2.1)	38(39.6)	74(77.1)	NA	NA
Lee^14^	2010	Korea	PCR+SEQ	173	2(1.2)	60(34.7)	117(67.6)	117(67.6)	NA
Kobayashi^19^	2011	Japan	PCR+SEQ/SSCP	581	5(0.9)	204(35.1)	NA	382(65.7)	124(21.3)
Marchetti^15^	2011	Italy	PCR+SEQ/HRMA	1046	37(3.5)	187(25.3)	542(73.3)	739(70.7)	218(29.5)
Paik^20^	2011	USA	MALDI-TOF MS	697	18(2.6)	452(65.8)	386(56.2)	NA	NA
An^21^	2012	China	HRMA	452	7(1.5)	NA	192(42.5)	307(67.9)	NA
Sasaki^22^	2012	Japan	PCR+SEQ	305	6(2.0)	148(56.7)	NA	NA	NA
Cardarella^5^	2013	USA	PCR+SEQ	883	36(4.1)	148(50.5)	229(78.4)	256(87.4)	237(80.9)
Ilie^23^	2013	France	PCR+SEQ	450	40(8.9)	158(35.1)	403(89.6)	NA	352(78.2)

Pts, patients; Mut BRAF, mutant BRAF; ADC, Adenocarcinoma; NA, not available; SEQ, sequencing; MALDI-TOF MS, matrix assisted laser desorption/ionization time of flight mass spectrometry; SSCP, single strand conformation polymorphism analysis; HRMA, high-resolution melting analysis.

### *BRAF* mutations and demographic and clinicopathologic characteristics of NSCLC

**Figure 2:**
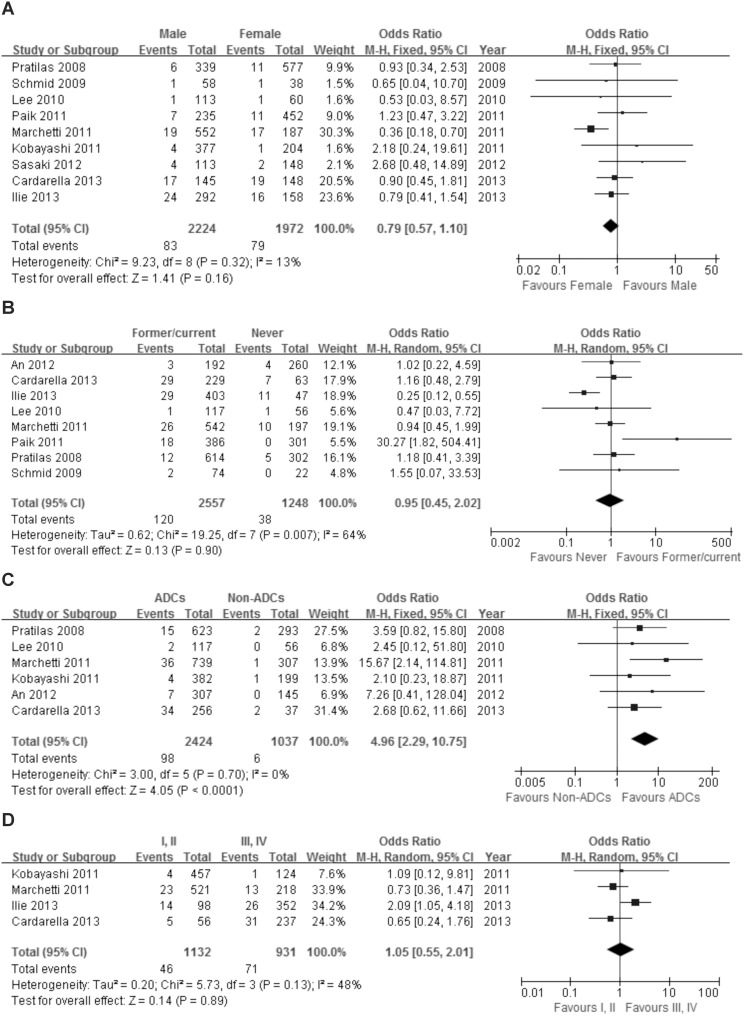
The association of BRAF mutations with gender (A), smoking (B), histology (C) and stage (D).

The pooled results of the association between *BRAF* mutations and demographic and clinicopathologic characteristics of NSCLC are reported in Table 2 and Figure 2. Nine studies presented data on the association between *BRAF* mutations and gender. *BRAF* mutations were detected in 83 of 2224 male patients (3.73%) and 79 of 1972 female patients (4.01%). There was no significant difference in the frequency of mutation by gender (OR = 0.79, 95%CI = 0.57–1.10). Data regarding the association between *BRAF* mutations and smoking was presented in eight studies. *BRAF* mutations were detected in 120 of 2557 former or current smokers (4.69%) and 38 of 1248 never smokers (3.04%). There was no significant difference in *BRAF* mutation rate in former or current smokers and never smokers (OR = 0.95, 95%CI = 0.45–2.02). Six studies reported the association between *BRAF* mutation and tumor histology. *BRAF* mutations were detected in 98 (4.04%) of 2224 ADC and 6 (0.58%) of 1037 non-ADCs. There were significant differences between ADCs and non-ADCs in *BRAF* mutation rate (OR = 4.96, 95%CI = 2.29–10.75). Only four studies contained information about clinical stage of NSCLC with *BRAF* mutations. *BRAF* mutations were detected in 46 of 1132 patients with stage I or II (4.06%) and 71 of 931 in patients with stage III or IV (7.63%). There were no significant difference in the *BRAF* mutation rate in stage I and II, and stage III and IV (OR = 1.05, 95%CI = 0.55–2.01).

**Table 2:** Association between BRAF mutation and gender, smoking, histology and stage in NSCLC.

Outcome	Mutant BRAF (%)	Statistical Method	Test of association		Heterogeneity test		
			OR (95%CI)	P	Chi^2^	I^2^	P
Gender							
Male	83/2224(3.73)	M-H, Fixed, 95%CI	0.79 [0.57, 1.10]	0.16	9.23	13%	0.32
Female	79/1972(4.01)						
Smoking							
Former/current	120/2557(4.69)	M-H, Random, 95%CI	0.95 [0.45, 2.02]	0.90	19.25	64%	0.01
Never	38/1248(3.04)						
Histology							
ADC	98/2424(4.04)	M-H, Fixed, 95%CI	4.96 [2.29, 10.75]	0.00	3.00	0%	0.70
Non-ADC	6/1037(0.58)						
Stage							
I, II	46/1132(4.06)	M-H, Random, 95%CI	1.05 [0.55, 2.01]	0.89	5.73	48%	0.13
III, IV	71/931(7.63)						

OR, Odds Ratio; CI, confidence interval.

### *BRAF* ^V600E^ mutation and demographic and clinicopathologic characteristics

**Figure 3:**
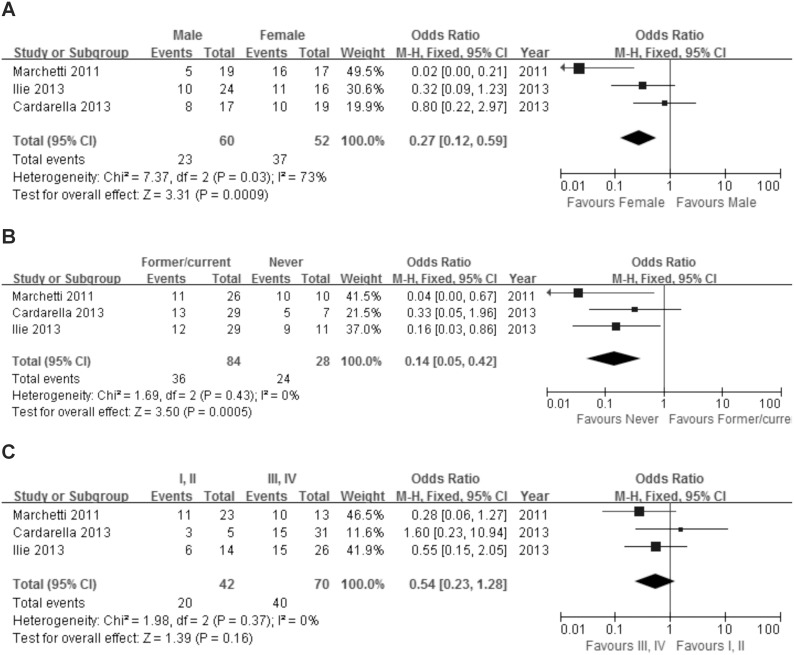
The association of BRAF^V600E^ mutations with gender (A), smoking (B) and stage (C).

The association of *BRAF* ^V600E^ mutation and demographic and clinicopathologic characteristics was evaluated (Figure 3). Three studies evaluating the *BRAF* ^V600E^ mutation in NSCLC were systematically analyzed using a fixed effects model. *BRAF* ^V600E^ mutations accounted for 53.6% (60/112) of all the *BRAF* mutations. *BRAF* ^V600E^ mutations were detected in 23 of 60 male patients (38.3%) and 37 of 52 female patients (71.2%). There was a significant differences in male and female expression of this mutation (OR = 0.27, 95%CI = 0.12–0.59). Of 84 former or current smokers, 36(42.9%) had *BRAF* ^V600E^ mutations. 24 of 28 never smokers (85.7%) had this mutation. The difference was significant between former or current smokers and never smokers (OR = 0.14, 95%CI = 0.05–0.42). *BRAF* mutations were detected in 20 of 42 patients with stage I or II (47.6%) disease and 40 of 70 in patients with stage III or IV (57.1%) disease. There was no significant difference in expression of this mutation in the two stage groups (OR = 0.54, 95%CI = 0.23–1.28).

### Subgroup analyses of *BRAF* mutations and demographic and clinicopathologic characteristics

When the combined studies included were stratified according to ethnicity, there were no statistically significant associations between *BRAF* mutation and gender, smoking history or stage, in agreement with the overall effect (Table S1). However, the OR of histology was 3.49 (95%CI: 0.79–15.45) for Asians, 5.75 (95%CI: 0.94–35.25) for non-Asians, and 3.59 (95%CI: 0.82–15.80) for mixed ethnicity. There was no statistically significant association between *BRAF* mutation and ADC, which was different from the overall effect (OR = 4.96, 95%CI = 2.29–10.75). Similarly, when the studies were grouped by the number of *BRAF* mutations, the combined OR for histology was 3.49 (95%CI: 0.79–15.45) for fewer mutations (<10). In this subgroup *BRAF* mutations were more frequently associated with ADCs. (Table S1).

### Sensitivity analysis and publication bias

Sensitivity analysis was conducted to ascertain whether modification of the inclusion criteria of this meta-analysis affected the final results. The sensitivity analysis revealed that none of the studies significantly affected the pooled ORs and CIs (data not shown). To investigate the presence of publication bias, a funnel plot of effects calculated from individual studies examining the association between *BRAF* mutations and demographic or clinicopathologic features was performed. There was no strong indication of publication bias among the studies included in this meta-analysis.

## Discussion

Studies of mutations of the *BRAF* gene have generated considerable interest because these mutations may be associated with increased sensitivity to agents directly targeting *BRAF* or *BRAF*-mediated downstream signaling pathways [25]. *BRAF* mutations have been analyzed using meta-analysis studies in melanoma, colorectal cancer and papillary thyroid cancer [26]–[29]. Mutations in the *BRAF* gene were closely related to particular demographic or clinicopathologic characteristics, including smoking habits, gender, clinical stage, differential and tumor histology. The effect of *BRAF* mutations on the clinical features of NSCLC have been reported for several years. A consensus has not been reached due to the small number of patients evaluated. We performed this meta-analysis to investigate the prevalence and characteristics of NSCLC patients with *BRAF* mutations in a large pooled sample of patients.

We systematically reviewed the literature describing the relationship between *BRAF* mutations and demographic or clinicopathologic features from 10 studies involving over 5500 patients with NSCLC. The rate of *BRAF* mutations was on average around 3% (170/5599), in agreement with previously published data [4], [7], [21]. The mutation rate from published studies varied from 0.9% to 8.9% [20], [24]. Ilie et al. explained the variation may be due to the exclusive evaluation of *BRAF* mutation in Caucasian patients with *EGFR*, *KRAS*, *PI3KCA*, *HER2* or *ALK* alterations [24]. Mutation rates of 2% and 3.5% were reported by Pratilas et al and Marchetti et al. in series comprised of 916 and 1046 patients, respectively [16], [19]. These were close to the frequency found in our study.

One of the aims of our study was to identify features that would help enrich patients for tumor mutation analysis. The meta-analysis was carried out to find an association between *BRAF* mutation and four clinicopathologic features. There were no significant association with gender and the incidence of *BRAF* mutation (OR = 0.79 95%CI = 0.57–1.10). An association between *BRAF* mutations and female has been reported in patients with colorectal cancer [29]–[31]. However, such an association has not been made for NSCLCs [5], [24], which corroborates with our results. Subgroup analyses by ethnicity finds a weak association with Asians having a slightly higher propensity for having *BRAF* mutation, but this was not statistically significant. Most of the studies also showed no association of *BRAF* mutation and smoking status. However, Paik et al reported that all patients with a *BRAF* mutation were current or former smokers [21]. However we see no such association in this analysis.

In recent years, scientists have made great progress toward understanding specific mutations of the cancer and targeting them with appropriate drugs [6]. Seidel et al. performed a great work with two cohorts studies including more than 6000 lung cancer patients, and demonstrated the association between lung tumor subtype and its predominant mutations, and the benefit of genetic testing and targeted therapy in these patients [6]. They found that most mutations showed histological subtype specificity and provided a blueprint for genomic diagnosis of lung tumors [6]. Therefore, we also performed the meta-analysis to found the association between BRAF mutation and histological subtype and clinical stage of NSCLC.

NSCLC is comprised of three different histologic types, squamous-cell carcinoma, large-cell carcinoma, and ADC. ADC accounts for more than 50% of all cases. We found that *BRAF* mutations as a whole were more common in ADCs than in other histologies (OR = 4.96 95%CI = 2.29–10.75), similar to a previous study [16]. There was no significant heterogeneity between studies (I^2^ = 0.0%, P = 0.70). An interesting finding of our study was that the pooled results in the subgroup stratified by ethnicity and number of mutations were significantly different from the overall effect of histology (Table S1). There was no significant difference in the rate of *BRAF* mutations and ADCs in the subgroup, while the differences were significant in the overall patient population. This may be explained by the number of cases included. When the number of patients was small in the subgroup, the association was not significant. When we investigated this association in a large number of patients, there was greater power to detect the association between *BRAF* mutation and ADC. This is the most valuable finding of this study. Clinical stage is an important factor in determining the prognosis of NSCLC. We found no significant association between low or high stage and *BRAF* mutation. Larger studies are needed to better examine this relationship.

Among the different mutations occurring in the *BRAF* gene, *BRAF* ^V600E^ is the most common [32]. So far three studies have investigated the association between *BRAF* ^V600E^ mutation and demographic or clinicopathologic features [5], [16], [24]. The number of V600E and non-V600E mutations detected was low, not allowing us to perform a separate analyse in other studies. The three studies found significant differences in the clinical features of patients with NSCLC with and without *BRAF* ^V600E^ [5], [16], [24]. Two reports found *BRAF* ^V600E^ mutations more frequent in females and never smokers, and not with any other clinicopathologic features [16], [24]. We also found the *BRAF* ^V600E^ mutation was significantly more frequent in women. The *BRAF* ^V600E^ mutation was also significantly more frequent in never-smokers compared to current or former smokers (OR = 0.14 95%CI = 0.05–0.42). Ilie et al reported that non-*BRAF* ^V600E^ mutations were significantly associated with early-stage tumours [24]. We did see a trend for earlier-stage disease but that was not statistically significant.

Heterogeneity is a potential problem that may affect the interpretation of all meta-analyses. To investigate the potential sources of heterogeneity that might modify effects of the *BRAF* mutations on smoking and stage, we performed subgroup analyses according to the ethnicity and number of *BRAF* mutations. The results in smoking and stage were not substantially changed, as indicated by subgroup and sensitivity analysis. Subgroup analysis by ethnicity demonstrated no clinical heterogeneity regarding the association between *BRAF* mutations and gender, smoking or stage. This indicates that overall estimation of the association between *BRAF* mutations and the clinical features of NSCLC is legitimate.

Our study had several limitations that need to be taken into consideration when interpreting the findings. Firstly, the number of included studies was small. More studies are needed to extend and confirm our results. Secondly, we did not collect data on the treatment and clinical outcomes of patients with *BRAF* mutations which will be done in a future study. Finally, we did not describe the association between BRAF mutation and smoking habit which grouped by former and current smokers separately due to lack of the data.

## Conclusions

Despite the limitations, our meta-analysis had some significant findings. We found that *BRAF* mutations were more frequent in ADCs, and were not associated with other histologic types. The BRAF^V600E^ mutation was significantly correlated with female and non-smoker NSCLC patients. The conclusions obtained here confirmed the reported association of BRAF mutations with specific demographic or clinicopathologic characteristics, which may be useful for the prediction of the NSCLC sub-types more accurately combined with other genetic changes.

## Supporting Information

Table S1 Subgroup analysis of the relationship between BRAF mutation and tumor characteristics according to ethnicity and number of mutations.
(DOCX)

Checklist S1 PRISMA 2009 Checklist.
(DOC)
