# Supplementary material for: BRAF Mutations in Patients with Non-Small Cell Lung Cancer: A Systematic Review and Meta-Analysis
Source: PLoS One. 2014 Jun 30;9(6):e101354. doi: 10.1371/journal.pone.0101354 (PMC4076330; doi:10.1371/journal.pone.0101354)
Supplement: Table S1 — Subgroup analysis of the relationship between BRAF mutation and tumor characteristics according to ethnicity and number of mutations. (DOCX) [file pone.0101354.s001.docx]

| **Table S1. S**ubgroup analysis of the relationship between BRAF mutation and tumor characteristics according to ethnicity and number of mutations. | | | | | | | | | | | | |
| --- | --- | --- | --- | --- | --- | --- | --- | --- | --- | --- | --- | --- |
| **Subgroup** | **Number of studies** | **Number of Pts** | **Statistical Method** | **Test of association** | | **Heterogeneity test** | | | | | |  |
|  |  |  |  | **OR (95% CI)** | P | **Chi²** | | **I^2^** | | **P** | |  |
| **Gender** |  |  |  |  |  |  | |  | |  | |  |
| Overall | 9 | 4196 | M-H, Fixed, 95% CI | 0.79 [0.57, 1.10] | 0.16 | 9.23 | | 13% | | 0.32 | |  |
| Ethnicity |  |  |  |  |  |  | |  | |  | |  |
| Asian | 3 | 1015 | M-H, Fixed, 95% CI | 1.87 [0.57, 6.17] | 0.3 | 0.98 | | 0% | | 0.61 | |  |
| Non-Asian | 5 | 2265 | M-H, Fixed, 95% CI | 0.71 [0.49, 1.02] | 0.06 | 5.80 | | 31% | | 0.21 | |  |
| Mixed | 1 | 916 | M-H, Fixed, 95% CI | 0.93 [0.34, 2.53] | 0.88 | NA | | NA | | NA | |  |
| Number of mutations |  |  |  |  |  |  | |  | |  | |  |
| ≥10 cases | 5 | 3085 | M-H, Fixed, 95% CI | 0.73 [0.52, 1.03] | 0.08 | 6.08 | | 34% | | 0.19 | |  |
| ＜10 cases | 4 | 1111 | M-H, Fixed, 95% CI | 1.60 [0.54, 4.74] | 0.39 | 1.43 | | 0% | | 0.70 | |  |
| **Smoking** |  |  |  |  |  |  |  | |  | |  | |
| Overall | 8 | 3805 | M-H, Random, 95% CI | 0.95 [0.45, 2.02] | 0.9 | 19.25 | | 64% | | 0.01 | |  |
| Ethnicity |  |  |  |  |  |  | |  | |  | |  |
| Asian | 2 | 625 | M-H, Fixed, 95% CI | 0.86 [0.23, 3.26] | 0.83 | 0.22 | | 0% | | 0.64 | |  |
| Non-Asian | 5 | 2264 | M-H, Random, 95% CI | 1.07 [0.34, 3.37] | 0.91 | 18.95 | | 79% | | 0.00 | |  |
| Mixed | 1 | 916 | M-H, Fixed, 95% CI | 1.18 [0.41, 3.39] | 0.75 | NA | | NA | | NA | |  |
| Number of mutations |  |  |  |  |  |  | |  | |  | |  |
| ≥10 cases | 5 | 3084 | M-H, Random, 95% CI | 1.02 [0.38, 2.73] | 0.97 | 19.05 | | 79% | | 0.00 | |  |
| ＜10 cases | 3 | 721 | M-H, Fixed, 95% CI | 0.96 [0.29, 3.18] | 0.94 | 0.34 | | 0% | | 0.84 | |  |
| **Histology** |  |  |  |  |  |  | |  | |  | |  |
| Overall | 6 | 3461 | M-H, Fixed, 95% CI | 4.96 [2.29, 10.75] | 0 | 3.00 | | 0% | | 0.70 | |  |
| Ethnicity |  |  |  |  |  |  | |  | |  | |  |
| Asian | 3 | 1206 | M-H, Fixed, 95% CI | 3.49 [0.79, 15.45] | 0.1 | 0.51 | 0% | | 0.78 | |  | |
| Non-Asian | 2 | 1339 | M-H, Random, 95% CI | 5.75 [0.94, 35.25] | 0.14 | 2.18 | | 54% | | 0.14 | |  |
| Mixed | 1 | 916 | M-H, Fixed, 95% CI | 3.59 [0.82, 15.80] | 0.09 | NA | | NA | | NA | |  |
| Number of mutations |  |  |  |  |  |  | |  | |  | |  |
| ≥10 cases | 3 | 2255 | M-H, Fixed, 95% CI | 5.51 [2.22, 13.66] | 0.0002 | 2.30 | | 13% | | 0.32 | |  |
| ＜10 cases | 3 | 1206 | M-H, Fixed, 95% CI | 3.49 [0.79, 15.45] | 0.1 | 0.51 | | 0% | | 0.78 | |  |
| **Stage** |  |  |  |  |  |  | |  | |  | |  |
| Overall |  |  | M-H, Random, 95% CI | 1.05 [0.55, 2.01] | 0.89 | 5.73 | | 48% | | 0.13 | |  |
| Ethnicity |  |  |  |  |  |  | |  | |  | |  |
| Asian | 1 | 581 | M-H, Random, 95% CI | 1.09 [0.12, 9.81] | 0.94 | NA | | NA | | NA | |  |
| non-Asian | 3 | 1482 | M-H, Random, 95% CI | 1.04 [0.48, 2.23] | 0.93 | 5.73 | | 65% | | 0.06 | |  |
| Number of mutations |  |  |  |  |  |  | |  | |  | |  |
| ≥10 cases | 3 | 1482 | M-H, Random, 95% CI | 1.04 [0.48, 2.23] | 0.93 | 5.73 | | 65% | | 0.06 | |  |
| ＜10 cases | 1 | 581 | M-H, Random, 95% CI | 1.09 [0.12, 9.81] | 0.94 | NA | | NA | | NA | |  |

Pts, patients; OR, Odds Ratio; CI, confidence interval
